# Effectiveness of control measures to prevent occupational tuberculosis infection in health care workers: a systematic review

**DOI:** 10.1186/s12889-018-5518-2

**Published:** 2018-05-25

**Authors:** Bey-Marrié Schmidt, Mark E. Engel, Leila Abdullahi, Rodney Ehrlich

**Affiliations:** 1 School of Public Health and Family Medicine, Faculty of Health Sciences, University of Cape Town, Falmouth Rd, Observatory, Cape Town, 7925 South Africa; 2 Department of Medicine, University of Cape Town, Cape Town, South Africa; 3 Vaccines for Africa, Institute of Infectious Disease and Molecular Medicine & Division of Medical Microbiology, University of Cape Town, Cape Town, South Africa; 4 Centre for Environmental and Occupational Health Research, School of Public Health and Family Medicine, University of Cape Town, Cape Town, South Africa

**Keywords:** Systematic review, Tuberculosis, Health care workers, Transmission control, Tuberculin skin test

## Abstract

**Background:**

A number of guideline documents have been published over the past decades on preventing occupational transmission of tuberculosis (TB) infection in health care workers (HCWs). However, direct evidence for the effectiveness of these controls is limited particularly in low-and middle-income (LMIC) countries. Thus, we sought to evaluate whether recommended administrative, environmental and personal protective measures are effective in preventing tuberculin skin test conversion among HCWs, and whether there has been recent research appropriate to LMIC needs.

**Methods:**

Using inclusion criteria that included tuberculin skin test (TST) conversion as the outcome and longitudinal study design, we searched a number of electronic databases, complemented by hand-searching of reference lists and contacting experts. Reviewers independently selected studies, extracted data and assessed study quality using recommended criteria and overall evidence quality using GRADE criteria.

**Results:**

Ten before-after studies were found, including two from upper middle income countries. All reported a decline in TST conversion frequency after the intervention. Among five studies that provided rates, the size of the decline varied, ranging from 35 to 100%. Since all were observational studies assessed as having high or unclear risk of bias on at least some criteria, the overall quality of evidence was rated as low using GRADE criteria.

**Conclusion:**

We found consistent but low quality of evidence for the effectiveness of combined control measures in reducing TB infection transmission in HCWs in both high-income and upper-middle income country settings. However, research is needed in low-income high TB burden, including non-hospital, settings, and on contextual factors determining implementation of recommended control measures. Explicit attention to the reporting of methodological quality is recommended.

**Trial registration:**

This systematic review was registered with PROSPERO in 2014 and its registration number is CRD42014009087.

## Background

Reviews in the past decade have concluded that health care workers (HCWS) in most countries have a higher tuberculosis (TB) disease incidence than the general population [1, 2], and further that HCWs in low-and middle-income countries (LMICs) have a higher prevalence and incidence of latent TB infection than their counterparts in high-income countries [2–4]. This gap is consistent with the occurrence of 80% of the global burden of TB in 22 LMICs, where overcrowded and under-resourced health care facilities are important sites of TB transmission, including multi-drug resistant TB [4, 5].

Since 1982 the US Centers for Disease Control and Prevention (CDC), and later the World Health Organisation (WHO), have published a number of TB transmission control guidelines for health care settings [6–10]. These guidelines classified protective practices under the now well-established headings of administrative, environmental and personal levels of protection. A fourth overarching category, “managerial activities”, at both national level and facility level, was elaborated in the WHO 2009 guideline [9].

Implementation of these guidelines in combination is credited with the control of nosocomial outbreaks of TB and particularly multidrug resistant TB in high-income countries such as the US (described below) and Italy [11], as well as an outbreak of extremely drug resistant TB in South Africa [12]. However, questions about the practicality of these guidelines in low resource health care settings was expressed as early as 1997 [13] and given expression in a WHO 1999 guideline [7]. A 2006 review deemed the evidence for a reduction in the risk of TB transmission after implementation of these measures to be “limited and weak” in LMIC settings, in contrast with stronger evidence from high-income countries [1]. Of ten control measures reviewed in the WHO 2009 Guideline (pp. 23–33) [9], nine were judged as having “low quality” evidence, the exception being a package of HIV-testing, isoniazid preventive therapy and access to anti-retroviral treatment for HCWs, which was supported by “high quality” evidence.

The need to identify more appropriate protective practices, i.e. fewer and less resource demanding, but nevertheless effective in these settings, has thus been frequently identified [4, 7, 13–15]. A 2007 systematic review [2] concluded that there was “limited evidence based on uncontrolled observations…that administrative controls are the most important component.” More recently, Nardell and others have argued for a refocusing of clinical administrative measures, namely FAST (“Find cases Actively, Separate temporarily and Treat effectively”) in health care settings where undiagnosed tuberculosis is likely to be main source of nosocomial infection for both staff and patients [13, 16].

Given the passage of a decade and the evolution of the methods of systematic review, we sought to update the evidence on the effectiveness of measures to control TB transmission in health care settings from the 2006 [1] and 2007 [2] reviews. We aimed to sharpen our understanding of the quality of evidence by using up to date methods of systematic review, including a detailed assessment of the risk of bias and overall quality of evidence.

The question we asked was as follows. Do tuberculosis transmission prevention practices in the categories of administrative, environmental and/or respiratory protection, collectively or individually, reduce the transmission of tuberculosis infection to HCWs? We sought to limit heterogeneity by concentrating on studies which used tuberculin skin test (TST) conversion as the most direct outcome indicator of transmission. A particular interest was LMIC settings, but the review was not limited to such studies. We restricted the review to longitudinal studies able to compare the same setting before and after implementation of an intervention so as to establish temporality and limit confounding.

## Methods

### Search strategy and study design

We considered all studies, observational or experimental in design, comprising HCWs, among whom TST conversion rates in both the “before” and “after” phases of implementation of control measures in the same facility could be measured. HCWs include nurses, doctors, laboratory staff, allied professionals and support staff. Studies were sought which compared either a single preventive measure, or multiple measures at one or different levels, against controls. A control was defined as non-use, less complete or less frequent use of TB transmission prevention measures. We excluded workplace studies of the effectiveness of disease screening for TB, or of screening for and treatment of latent TB infection in preventing disease.

The electronic searches were conducted to the end of July 2017. We developed a search strategy comprising relevant medical subject headings (MeSH) and keywords relating to tuberculosis, HCWs, and tuberculosis control measures in Medline, shown in Appendix. The search strategy included a combination of the following keywords: TB, tuberculosis, mycobacterium, health personnel, hospital personnel, respiratory protective device, mass screening, education, triage, patient isolation, early diagnosis, risk assessment, guideline, policy and controlled environment.

We translated the Medline search strategy into Scopus, Trip, LILACS, Cochrane Central Register of Controlled Trials, World Health Organisation International Clinical Trials Registry Platform, and ClinicalTrials.gov, while making the necessary vocabulary adjustments. We placed no limitations on date or language. Both published and unpublished studies were considered. We looked over the reference lists of identified studies for additional studies. Authors and experts in the field were contacted for unpublished and published work. A manual internet search, using Google, was performed at the end of the systematic search to identify grey literature.

### Data extraction and analysis

BS and ME verified whether the relevant articles met the inclusion criteria. This was checked by a third reviewer, RE, and any disagreements resolved through discussion. BS extracted relevant data items using a standardised form, which was checked by ME and RE.

### Assessment of risk of bias and quality of evidence.

Risk of bias in the included studies was assessed using criteria from the Cochrane Effective Practice and Organisation of Care (EPOC) Group [17]. These criteria are designed to assess the risk of bias in research focused on the delivery, practice and organisation of health care services. The criteria are relevant to controlled trials and controlled before-after studies. The quality of the evidence was assessed using GRADE criteria [18].

## Results

### Study selection

**Fig. 1:**
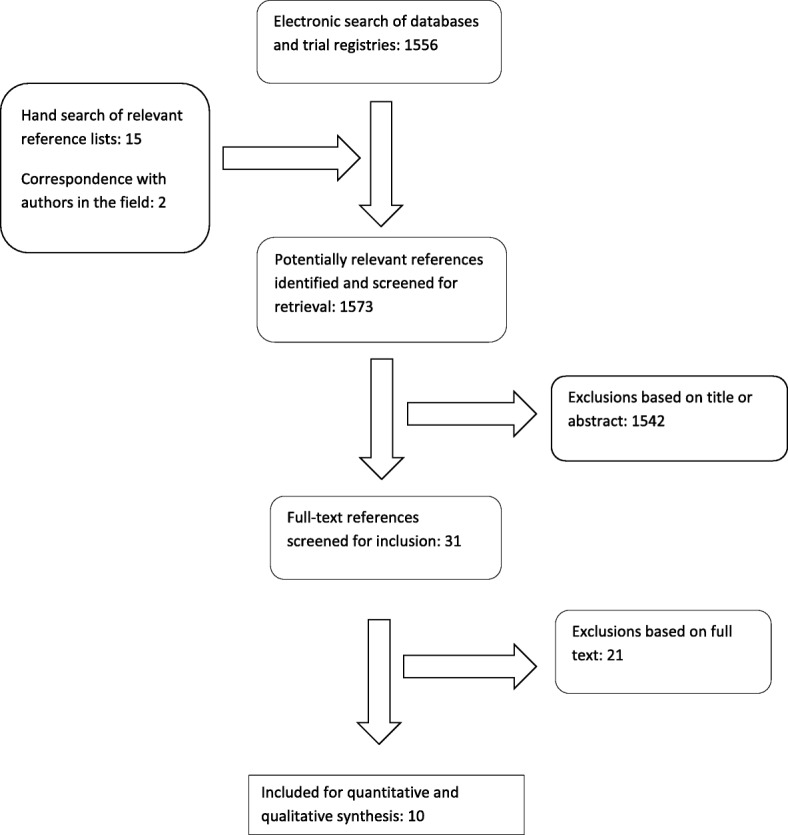
Selection of studies for review

Figure 1 shows a flow diagram of the search results. We screened 1573 references, i.e. titles and abstracts, from which 31 full text articles were deemed potentially eligible. We did not find non -English titles. After scrutiny of the full text, ten articles met our inclusion criteria. Of the 21 *excluded* articles, only eight [19–26] were studies of TST conversion or incident TB cases in a health care worker sample following transmission control measures. These eight excluded studies are listed in Table 1, with reasons for exclusion and findings. Three were from LMICs, namely Brazil, South Africa and Malawi. Malawi was the only study found from a low income country, but was not included in the review because it compared TB disease rates, rather than of TB infection, our primary outcome. This study found a slight but non-significant decrease in TB disease incidence after introduction, but incomplete implementation, of infection control guidelines.

**Table 1:** Excluded studies of tuberculin skin test (TST) conversion rates or TB disease in HCWs and TB transmission control measures (*n* = 8)

First author, year (country)	Reason for exclusion	Relevant finding
High-income countries		
Fridkin, 1995 [19] (USA)	Cross-hospital survey of TST conversion rates.	Conversion rates lower in hospitals with transmission control measures.
Fella, 1995 [20] (USA)	Overlap with Louther et al. [32] (Same hospital, similar period of study, 1991–1993)	Decline in proportion of TST conversions over six 6-month cycles (20.7 to 5.8%) while CDC guidelines implemented.
Holton, 1997 [21] (Canada)	Cross-hospital survey of TST conversion rates.	Compliance with transmission control measures inadequate in both high and low TB risk facilities.
Boudreau 1997 [22] (USA)	Longitudinal study of TST conversion rates but authors unable to attribute decline to transmission control measures.	TST conversion rates fell over time in TB exposed health care workers.
Tokars, 2001 [23] (USA)	Two-hospital study of TST conversion rates.	Very low rates of TST conversion in both hospitals.
Low-and middle-income countries		
Harries, 2002 [24] (Malawi)	TB case notification rates before and after infection guidelines introduced.	Small non-significant decline in TB case notification rates (3.7 to 3.2%).
Roth, 2005 [25] (Brazil)	Cross-hospital survey of TST conversion rates.	Conversion rates lower in hospitals with transmission control measures.
O’Hara, 2017 [26] (South Africa)	Cross-sectional ecological study of TB incidence rates.	TB incidence negatively associated with overall infection control score. Of specific components, only use of respirators remained protective after multivariable adjustment.

*CDC* Centers for Disease Control and Prevention

All ten *included* studies were observational [15, 27–35], comparing the risk of TST conversion among HCWs during periods before and after control measures were implemented. Descriptions of the included studies are presented in Table 2. The ten studies had sample sizes (before/after) ranging from 25/27 to 3579/5153 participants. Five of the studies [15, 31, 32, 34, 35] reported TST conversion rates (per person-time), of which two [15, 35] did not provide count data. The remaining five studies [27–30, 33] provided proportions of staff converting during the before and after stages. Only one study investigated the effect of a single level of intervention (engineering controls); control measures at more than one level were studied in the rest [33].

**Table 2:** TB transmission control measures and tuberculin skin test (TST) conversion: study details (*n* = 10)

First author, year. Country, period of study.	Sample size. Type of health care worker; Location (B = before, A = After intervention)^a^	Transmission control measures (Guidelines referenced as basis for intervention)	Cycles of observation (B = before, A = After intervention)^a^	PPD, dose. (1- step test unless otherwise indicated) Definition of conversion (induration diameter) (TU = tuberculin units)
High-income settings (all USA)				
Blumberg 1995. [27] USA, 1992–1994.	3579 (B) and 2975–5153 (A1-A4). HCWs; hospital, Atlanta	Administrative: Expanded and stricter respiratory isolation regimen. TB transmission control nurse. Expanded staff TB education. Environmental: Rooms with negative pressure for respiratory isolation. Personal: Submicron respirators for HCWs in isolation rooms. (CDC 1990, 1994)	5 cycles: B: 6 months A1: 6 months A2: 6 months A3: 6 months A4: 6 months	Aplisol or Tubersol, 5 TU. Conversion: > 10 mm following prior negative test.
Jarvis, 1995. [28] USA, 1989–1992.	Intervention: 152 (B) and 173 (A). HCWs; 2 hospitals, Miami, New York. Controls: 22 (B) and 33 (A). HCWs; third hospital, New York.	Administrative: Education of staff for earlier recognition, diagnosis and rapid isolation of TB patients. Restriction of patient movement. Expanded TB drug regimen. Environmental: Increase in number of AFB isolation rooms. Negative pressure in these rooms. Negative pressure ventilated booths for aerosol generating procedures. Personal: Respirators and submicron surgical masks. (CDC 1994)	B: Up to 24 months A: Not given	PPD not specified. Conversion: > 10 mm (baseline unknown) or > 10 mm *increase* in induration (baseline known).
Maloney, 1995. [29] USA, 1990–1992.	Intervention: 90 (B) and 78 (A). Staff on wards with TB patients; hospital, New York. Controls: 254 (B) and 228 (A). Staff on all other wards.	Administrative: Improved test based AFB isolation on admission. Expanded treatment regimen. More efficient and quicker laboratory diagnosis. Drug sensitivity probe added. Environmental: AFB rooms with negative pressure. Portable chambers for cough inducing procedures. Personal: Change from non-moulded to moulded surgical masks. (CDC 1990).	B: 18 months A: 14 months	PPD not specified. Conversion: > 10 mm with negative prior test.
Wenger, 1995. [30] USA, 1990–1992.	25 (B) and 27 (A). HCWs in HIV ward; hospital, Miami.	Administrative: Stricter respiratory isolation regimen. Sputum induction only in isolation rooms. Additional laboratory staff with faster results turnaround. Environmental: Automatic door closers in isolation rooms. Negative pressure isolation rooms and HIV ward. Aerosolised pentamidine in TB isolation rooms. Personal: Surgical submicron masks worn in isolation rooms. Dust-mist particulate respirators (late intervention). (CDC 1990)	3 cycles: B: 5 months A1: 9 months A2: 16 months	PPD not specified. 5 TU. Conversion: > 10 mm and at least 6 mm *increase* over previous induration.
Bangsberg, 1997. [31] USA, 1992–1994.	126 (B1-B2) and 124–138 (A1-A3). Medical house staff; hospital, New York.	Administrative: Isolation of high risk patients. Specialised TB service for patients with HIV or known HIV risk factors and suspicion of pneumonia. Environmental: Negative pressure rooms. UVGI in emergency and patient care areas. Personal: Instruction and fit-testing on respirators to be worn by staff in care of isolated patients. (CDC 1993)	5 cycles: B1:11–35 months B2: 6 months A1: 6 months A2: 6 months A3: 6 months	Aplisol or Tubersol, 5 TU. Conversion: ≥6 mm *increase* to a value of at least 10 mm.
Louther, 1997. [32] USA, 1991–1994.	898 (B) and 971 (A). HCWs; hospital, New York.	Administrative: Early respiratory isolation of suspected TB cases.^b^ Environmental: Negative pressure rooms. UVGI. Personal: Face shield masks. Particulate and dust-mist-fume respirators. (CDC 1994)	B^c^: 24 months A: 24 months	Aplisol, 5 TU 1991–1992; Tubersol, 5 TU 1993–1994. (2-step tested employees excluded; tests by outside physicians allowed.) Conversion: > 10 mm *increase* over baseline within 2 years. ^b^Interventions assumed to be same as described in Fella et al.[20] (See Table 1). ^c^“Before” period simultaneous with introduction of intervention.
Behrman, 1998. [33] USA, 1993–1996.	Intervention: 50 (B) and 64 (A). Emergency Department employees; hospital, Philadelphia. Controls: 2514 (B) and 3000 (A). Other hospital employees.	Administrative: None. Environmental: Respiratory isolation rooms meeting CDC standards. Improved ventilation with at least 25% fresh air in the Emergency Department. 100% non-recirculated air in Trauma area. Laminar flow away from registrars with droplet shields. Personal: None. (CDC 1990)	B: 12 months A: 12 months	PPD not specified. 5 TU. Conversion: > 10 mm following prior negative test (< 5 mm).
Blumberg, 1998. [34] USA, 1992–1997.	2144 (B) and 2123 (A). Rotating house staff (residents and fellows); hospital, Atlanta.	Administrative: Expanded and stricter respiratory isolation regimen. TB transmission control nurse. Expanded staff. TB education. Environmental: Rooms with negative pressure for respiratory isolation. Personal: Submicron respirators for HCWs in isolation rooms. (CDC 1994)	B: 6 months A: 54 months	Aplisol or Tubersol, 5 TU. Conversion: > 10 mm following prior negative test.
Lower and middle-income settings				
Yanai, 2003. [35] Thailand, 1995–1999.	369 (B) and 164 (A1). HCWs; hospital, Chaing Rai.	Administrative: Training of HCWs on TB transmission prevention. Faster case detection, TB diagnosis, treatment initiation and isolation. Infectious patients trained in cough and mask practice. One-stop outpatient TB service with faster throughput and referral out. Environmental: TB isolation rooms with negative pressure attached to wards. Increased natural ventilation in high risk wards. Safety cabinets, air exhaust and ultraviolet air disinfection in laboratory. Personal: N95 respirators for HCWs. Air purified respirators with high efficiency particulate filters for laboratory staff. (CDC 1994)	3 cycles: B: 24 months (1995 to 1997) A1: 12 months A2: 12 months	Tubersol, 5 TU. 2-step test. Conversion: > 10 mm *increase* over negative prior test (< 10 mm).
da Costa, 2009. [15] Brazil, 1998–2003.	406 (B) and 193 (A). HCWs; hospital, Rio De Janeiro.	Administrative: Increased respiratory isolation, rapid diagnosis. Environmental: None. Personal: Worker education in use of respirators. (Guidelines not specified)	B: 23 months A: 16 months	PPD not specified. 2-step where possible. Conversion: > 10 mm *increase* over previous TST in 2-step; or > 15 mm *increase* in 1-step.

*PPD* purified protein derivative, *AFB* acid fast bacillus, *CDC* Centers for Disease Control and Prevention, *UVGI* ultraviolet germicidal irradiation, *HCWs* health care workers

^a^B1, B2, A1, A2, etc. if more than two cycles

^b^Interventions that are assumed to be same as described in Fella et al. 20 (See Table 1).

^c^The "before" period is simultaneous with the introduction of intervention

### Summary of results

Results are presented in Table 3. All the studies found a substantial decline in the rate of TB conversion per 100 person-years of observation or in the proportion of HCWs showing conversion. Among those which provided rates [15, 31, 32, 34, 35], the size of the reduction varied, with the smallest effect (comparing the last to the first period when there were multiple periods) being 35% [15]. In three of the five studies in which proportions were compared [28–30] the before and after periods differed in length [29, 30] or were not specified [28]. With this qualification, the proportion of staff converting in these studies was substantially lower in the after period, consistent with the rate reductions reported above. One study from the USA which provided counts [18] did not specify the before and after periods of observation; the reported conversion proportions fell from 24.1 to 0% and 12.2 to 3.3% in the two intervention hospitals.

**Table 3:** TB transmission control measures and tuberculin skin test (TST) conversion: Risk of bias assessment and results (*n* = 10)

Studies	Risk of bias							Results		Notes
First author, year.	Similar baseline outcome measures	Similar baseline characteristics	Incomplete outcome data (attrition bias)	Blinding or objective assessment of outcome	Protected against contamination	Selective outcome reporting (reporting bias)	Selection and confounding bias	Conversion rate per 100 person years or proportion converting (%) (B = before, A = after)	Relative rate or proportion (A/B)	B1, B2, A1, A2, etc., if more than two cycles.
Studies reporting *rates* of conversion										
Bangsberg, 1997 [31]	N/A	N/A	Unclear	Low	N/A	Low	High	B1: 5.8	B1:1.00	
								B2: 5.1	B2: 0.88	
								A1: 0.0	A1: 0.00	
								A2: 2.3	A2: 0.40	
								A3: 0.0	A3: 0.00	
Louther, 1997 [32]	N/A	N/A	Unclear	Low	N/A	Low	High	B: 7.2 (65/898)	B: 1.00	Fella et al. [20] describing same study, mention possible overestimate of conversions in before period.
								A: 3.3 (32/971)	A: 0.46	
Blumberg, 1998 [34]	N/A	N/A	Unclear	Low	N/A	Low	High	B: 5.98	B: 1.00	
								A: 1.09	A: 0.18	
Yanai, 2003 [35]	N/A	N/A	Unclear	Low	N/A	Low	Low		Univariate:	Multivariate adjustment for sex, age, area TB risk, BCG scar, duration of work (≤ 12 vs > 12 months) and frequency of patient contact. ^a^Discrepancy between rates and rate ratios in Table 2 of article – figures here are recalculations.
								B: 9.3	B: 1.00	
								A1: 6.4	A1: 0.69^a^	
								A2: 2.2	A2: 0.24^a^ Multivariate: A1: 0.40 A2: 0.01	
da Costa, 2009 [15]	N/A	N/A	High	Low	N/A	Low	High	B: 4.8^c^	B: 1.00	^c^Converted from person-months to person-years. High loss to follow up reported.
								A: 3.1	A: 0.65	
Studies reporting only *counts,* i.e. proportions converting in each period										
Blumberg, 1995 [27]	N/A	N/A	Unclear	Low	N/A	Low	High	B: 118/3579 (3.3%)	1.00	Each period 6 months.
								A1: 51/2975 (1.7%)	0.51	
								A2: 67/4715 (1.4%	0.42	
								A3: 30/4775 (0.6%)	0.18	
								A4: 23/5153 (0.4%)	0.12	
Jarvis, 1995 [28]	High	Unclear	High^d^	Low	Low	Low	High	Intervention groups: Hospital “A”		^d^Authors reported insufficient TST data from control hospital. After period unspecified.
								B: 7/29 (24.1%)	1.00	
								A: 0/23 (0%)	0.00	
								Hospital “D”		
								B: 15/123 (12.2%)	1.00	
								A: 5/150 (3.3%)	0.27	
								Control: Hospital “B”		
								B: 2/22 (9.1%)	1.00	
								A: 6/33 (18.2%)	2.00	
Maloney, 1995 [29]	High	Unclear	Unclear	Low	Unclear	Low	High	Intervention group: Wards with TB patients	–	^e^Periods differed in duration: 18 vs 14 months. Before proportion standardised to 14 months.
								B: 15/90 (16.7%) 12^e^/90 (13.3%)	- 1.00	
								A: 4/78 (5.1%)	0.38	
								Controls: Other wards		
								B: 7/254 (2.8%)	1.00	
								A: 9/228 (3.9%)	1.39	
Wenger, 1995 [30]	N/A	N/A	Unclear	Low	N/A	Low	High	B: 7/25 (28%)	1.00	Periods differed in duration: 5 vs 9 and 16 months. Given zero count in A2, no period adjustment needed.
								A1^:^ 3/17 (17.6%)	0.63	
								A2: 0/23 (0%)	0.00	
Behrman, 1998 [33]	High	Low	Unclear	Low	Unclear	Low	Low	Intervention group: Emergency Department		Each period 12 months. Possible co-intervention via increased staff vigilance regarding early diagnosis and use of respirators.
								B: 6/50 (12%)	1.00	
								A: 0/64 (0%)	0.00	
								Controls: Other hospital employees		
								B: 51/2514 (2.0%)	1.00	
								A: 36/3000 (1.2%)	0.60	

*N/A* not applicable

Only two of the ten studies were conducted in LMICs [15, 35], Thailand and Brazil, which are classified as upper middle within this category, while the remaining eight were conducted in one high-income country, the USA [27–34]. The Thai study showed a reduction from 9.3 to 2.2 per 100 person-years [35]. It was also the only study among the ten to control confounding with multivariate analysis, which resulted in an even larger reduction. The Brazilian study [15] recorded a decline from 4.8 to 3.1 per 100 person-years. However, the TB incidence rate in HCWs almost doubled over the course of the study, despite the fall in incident TB infection rates [15].

### Assessment of risk of bias

The risk of bias of the three studies with “control” participants [28, 29, 33] was assessed using all seven EPOC criteria as detailed in Table 3. Risk of bias was scored low for blinding/objective assessment of outcome and selective outcome reporting. There were large differences in the baseline outcome measure between the intervention and control groups in all three, reflecting different baseline facility TB exposure risks. In only one study [33] was the comparability of intervention and control groups clearly stated. The other two [28, 29] were classified as having high risk of confounding or selection bias.

Only the five criteria applicable to studies lacking control groups could be used to assess the risk of bias for the remaining seven studies [15, 27, 30–32, 34, 35] (Table 3). Uncontrolled studies are particularly vulnerable to confounding, i.e. a change in TB conversion rates over time due to factors unrelated to the control measures introduced, such as a decline in the case load of patients with tuberculosis seen at the facility. All seven were scored as having low risk of bias for blinding/objective assessment of outcome and selective outcome reporting, and six [15, 27, 30–32, 34] with high risk for selection and confounding bias. Only one study [35] adjusted for potential confounders of the difference between before and after intervention phases and could be scored as having low risk of confounding bias.

There were other sources of unmeasured variation. In only one study [32] was it reasonably clear that the same individuals were studied across the before and after periods. The remaining studies reported different numbers of participants (allowing for exclusion of baseline period converters) in the before and after periods, with no information about what proportion of individuals were the same at follow up. This may introduce potential confounding across periods if, for example, susceptibility to conversion is highest in the first year of exposure [35]. Finally, the method of conducting the TST varied across studies (and within some studies), with different tuberculin products and definitions of conversion and the use of a two-step test procedure in some but not others. These sources of variability may have introduced further heterogeneity between studies and even within some studies.

### Quality of the evidence

There were a number of features of the evidence in favour of effectiveness: a) consistency of findings (b) large effect sizes (c) sustained decline in conversion in studies which included multiple “after” periods (Table 3) and (d) even greater risk reduction in the studies scored as low risk for confounding [35]. However, given that only observational studies were included in this review and the high or unclear risk of bias on at least some EPOC criteria in all ten studies, the overall quality of the studies reviewed was classified as “low” on GRADE criteria.

## Discussion

### Overall findings

There is consistent but low-quality evidence that combined control measures at the various levels in line with CDC recommendations are effective in preventing transmission of TB in HCWs. Eight of the ten studies were conducted in the USA, a low TB burden high-income country, in the 1990s in response to the rise of nosocomial TB and the appearance of multidrug resistant tuberculosis. All of these studies were conducted between 1989 and 1997 (Table 2).

Only two studies done in LMICs were found which met the inclusion criteria of the review - one in Thailand [35] and one in Brazil [15], both high TB burden, upper-middle-income countries. These showed a strong preventive effect of the intervention, no different from those in the high- income country studies. The Brazilian study of 2009 is the only study found from the last decade. Research on the effectiveness of TB control measures in high TB burden countries, many at lower resource levels than Thailand and Brazil, thus remains scarce, a conclusion unchanged over the course of a decade [1, 2].

Introduction of the CDC “package” of control measures was the norm. Only one study could be found which assessed the effectiveness of a single level of TB intervention, i.e. environmental, which was found to be protective [33]. In a number of the studies [27, 28, 30, 31] the authors suggested that administrative controls were primarily responsible for the decline in conversion risk, particularly as they were introduced earlier than other levels. Only one study [15] explicitly targeted administrative measures although in combination with personal respiratory protection. Only one study [29] explicitly listed all the elements that make up the current FAST package including drug sensitivity testing, although most of the studies included respiratory isolation as an administrative control with rapid diagnosis being part of a number of others (Table 2). All of the studies were conducted in hospitals, a bias which might be appropriate to the USA but not to LMICs, where district level services, including primary care facilities, are the first contact with patients and provide a large share of TB care [8].

### Quality of review and of evidence

We aimed to minimise review bias by conducting comprehensive searches without date or language restrictions. In our protocol, we made provision for various study designs suitable for assessing the effect of TB control measures on risk of TB infection in HCWs. None of the studies found were randomised controlled trials, which is understandable given that randomisation of a hospital or wards is impractical, logistically challenging and/or costly. Thus, all included studies appeared to be “naturalistic” in that the research was conducted in parallel with operational implementation of TB interventions. Although five of the ten studies [15, 28–30, 34] made some reference to implementation, it was not possible to judge the degree of implementation. However, inadequate implementation would be more likely to produce negative studies than the consistently positive studies found. Unlike the previous reviews, we excluded cross-sectional studies comparing different facilities [25] because of the high risk of confounding, as well as efficacy studies in highly controlled settings, e.g. Dharmadhikari et al. [36], and modelling studies, e.g. Basu et al. [37].

Taking the EPOC criteria as our starting point, the risk of bias in almost all of the studies was assessed overall as high or at best unclear. We concluded with the GRADE definition of “low quality evidence”, namely, that “further research is very likely to have an important impact on our confidence in the estimate of effect and is likely to change the estimate” (Table 4).

**Table 4:** Summary of findings on the quality of evidence

**Population:** Health care workers **Settings:** USA [eight studies], Brazil [one study] and Thailand [one study] **Intervention:** Administrative, environmental and personal preventative measures **Comparison:** Non-use, less frequent or less intense use of TB transmission control measures			
**Outcomes**	**Impacts**	**No of Participants** **(studies)**	**Quality of the evidence** **GRADE)**
**Tuberculin Skin Test (TST) conversion**	All studies showed a decrease in TST conversion	7 839^a^ before intervention; 9 084^a^ after intervention (10 studies)	⊕ ⊕ ⊝⊝ Low
* We downgraded the quality of evidence because of the uncontrolled (observational) study design and unclear/high risk of bias in most of the included studies. **GRADE Working Group grades of evidence:** **High quality:** Further research is very unlikely to change our confidence in the estimate of effect. **Moderate quality:** Further research is likely to have an important impact on our confidence in the estimate of effect and may change the estimate. **Low quality:** Further research is very likely to have an important impact on our confidence in the estimate of effect and is likely to change the estimate. **Very low quality:** We are very uncertain about the estimate.			

^a^If more than one before or after period, higher or highest number used for each phase. Does not include controls

### Recommendations

There is a pressing need for further research on the prevention of transmission of TB infection in high TB burden low income settings, taking into account the evolution of thinking since the influential CDC 1994 guidelines, and specifically prioritisation of elements appropriate to the resources and capabilities of the health systems with high MDR TB and HIV burdens. Administrative measures such as FAST [16] are the current focus in this regard, particularly given the pressures for decentralised care to manage MDR TB [38]. Ventilation solutions appropriate to building design and climate in high patient throughput settings are also under-researched [39]. Research settings should include both hospitals and district or primary health care services as well as special locations such as laboratories. Missing from the literature are studies of “upstream” or health system factors which may determine the successful application of facility level control measures, such as political commitment, leadership, funding, or information systems [40].

To strengthen the quality and presentation of such research, studies with more explicit methodological quality than those reviewed are needed. Although randomised controlled trials are desirable for reducing selection bias and confounding, controlled before and after studies may have to be relied upon, but with greater attention to the comparability of before and after intervention groups, the time periods being compared and intervention and control groups where relevant. An alternative approach is the study of quality improvement interventions for intermediate processes, such as the steps that make up FAST [41]. However, ultimately the effectiveness in preventing TB infection in HCWS needs be tested directly [42]. The outcome indicator used in the current review, namely TST conversion, remains the most practical measure for studies in LMICs. There is as yet insufficient evidence of the reliability of repeated interferon gamma release assays as an indicator of interval infection in these settings [43]. Use of incident tuberculosis as the outcome is constrained by variability in susceptibility to progress to active disease, which might dilute a short-term association between transmission control measures and incident TB.

Assessment of bias should be explicit, using an accepted schema. However, even before-after studies are logistically demanding, requiring establishment of an accurate baseline rate of conversion, proper implementation of the intervention, and subsequent follow up periods of measurement. Sustained and accurate record-keeping [44], consistency of testing practice, assessment of co-intervention and confounding over this period all increase the resource and staffing requirements of such research.

## Conclusion

This review has found very little progress over the past decade in updating the evidence, specifically in high TB burden low income settings, on the effectiveness of recommended practices to prevent nosocomial transmission of TB infection in health care workers. The reasons for this lack of progress were not investigated, but barriers are likely to include the many practical difficulties in conducting such research.

In the face of the continuing TB epidemic in many countries, such studies deserve a higher priority in funding programmes than has been the case over recent decades. Such funding should encourage research in a breadth of settings, inclusion of systems or contextual elements in the intervention, and management and reporting of quality issues inherent in different study designs.

## Appendix

**Table 5:** Search strategy developed in PubMed database

#1	Search ((TB) OR Tuberculosis) OR Mycobacterium
#2	Search (health personnel) OR hospital personnel
#3	Search respiratory protective device
#4	Search mass screening
#5	Search education
#6	Search triage
#7	Search patient isolation
#8	Search early diagnosis
#9	Search risk assessment
#10	Search guideline
#11	Search policy
#12	Search controlled environment
#13	Search ((((TB) OR Tuberculosis) OR Mycobacterium)) AND ((health personnel) OR hospital personnel)
#14	Search ((((((TB) OR Tuberculosis) OR Mycobacterium)) AND ((health personnel) OR hospital personnel))) AND respiratory protective device
#15	Search ((((((TB) OR Tuberculosis) OR Mycobacterium)) AND ((health personnel) OR hospital personnel))) AND mass screening
#16	Search ((((((TB) OR Tuberculosis) OR Mycobacterium)) AND ((health personnel) OR hospital personnel))) AND education
#17	Search ((((((TB) OR Tuberculosis) OR Mycobacterium)) AND ((health personnel) OR hospital personnel))) AND triage
#18	Search ((((((TB) OR Tuberculosis) OR Mycobacterium)) AND ((health personnel) OR hospital personnel))) AND patient isolation
#19	Search ((((((TB) OR Tuberculosis) OR Mycobacterium)) AND ((health personnel) OR hospital personnel))) AND early diagnosis
#20	Search ((((((TB) OR Tuberculosis) OR Mycobacterium)) AND ((health personnel) OR hospital personnel))) AND risk assessment
#21	Search ((((((TB) OR Tuberculosis) OR Mycobacterium)) AND ((health personnel) OR hospital personnel))) AND guideline
#22	Search ((((((TB) OR Tuberculosis) OR Mycobacterium)) AND ((health personnel) OR hospital personnel))) AND policy
#23	Search ((((((TB) OR Tuberculosis) OR Mycobacterium)) AND ((health personnel) OR hospital personnel))) AND controlled environment

Search strategy developed in PubMed database. A search strategy was developed in the PubMed database comprising relevant medical subject headings (MeSH) and keywords, such as tuberculosis, HCWs, and tuberculosis control measures

## Abbreviations

CDC: Centers for Disease Control and Prevention
EPOC: Effective Practice and Organisation of Care
FAST: “Find cases Actively, Separate temporarily and Treat effectively”
HCWs: Health care workers
LMICs: Low-and middle-income countries
MeSH: Medical subject headings
TB: Tuberculosis
TST: Tuberculin skin test
WHO: World Health Organisation
